# ELF5 modulates casein synthesis in goat mammary epithelial cells via JAK2/STAT5 signaling pathway

**DOI:** 10.5713/ab.25.0181

**Published:** 2025-10-22

**Authors:** Yuzhu Guo, Cunxia Ma, Tianxiang Meng, Tongtong Tu, Shuangshuang Cui, Yinghui Ling, Yunhai Zhang, Hongyu Liu, Ning Song

**Affiliations:** 1 Anhui Province Key Laboratory of Local Livestock and Poultry Genetic Resource Conservation and Germplasm Innovation, College of Animal Science and Technology, Anhui Agricultural University, Hefei, China

**Keywords:** Casein Synthesis, E74-like Factor 5, Goat Mammary Epithelial Cells, Signal Transducer and Activator of Transcription 5

## Abstract

**Objective:**

Goat milk contains a high concentration of caseins which are beneficial to human health. Understanding the regulatory mechanisms of casein synthesis in goats contribute to improving milk quality. Epithelial-specific E74-like factor 5 (ELF5) is important in milk protein synthesis, although the molecular mechanism by which ELF5 regulates casein synthesis remains unclear. This study aims to investigate the regulatory roles of ELF5 on casein production in goat mammary epithelial cells (GMECs).

**Methods:**

Primary GMECs were isolated from goats and characterized using immunofluorescence and oil red O staining. The goat *ELF5* gene overexpression vector and small interfering RNA were transfected into GMECs, respectively. Cell viability was accessed using cell counting kit-8 assay, and cell apoptosis was detected by flow cytometry. Genes involved in cell proliferation, apoptosis, and casein synthesis were examined by quantitative real-time polymerase chain reaction and Western blot. After co-treatment of Janus kinase 2 (JAK2) or signal transducer and activator of transcription 5 (STAT5) inhibitors, casein gene expression was examined by using Western blot. The interaction between ELF5 and STAT5 was verified by co-immunoprecipitation assay.

**Results:**

ELF5 enhances cell viability and the expression of genes associated with proliferation, while simultaneously inhibiting apoptosis and the expression of apoptosis-related genes. Then, ELF5 upregulates the expression of α_S1_-, α_S2_-, β- and κ-casein, in addition to enhancing JAK2/STAT5 signaling pathway. The inhibition assay of JAK2 and STAT5 activity reveals that ELF5 regulates casein synthesis via JAK2/STAT5 signaling pathway.

**Conclusion:**

ELF5 upregulates casein synthesis through the activation of the JAK2/STAT5 signaling pathway, offering a strategy for manipulating ELF5 to increase casein content and improve the protein quality of goat milk.

## INTRODUCTION

Goat milk protein contains essential amino acids and is an important source of dietary protein. Milk protein consist of casein and whey protein, with casein accounting for 80% of the total protein in goat milk. Casein is divided into αS1-casein (CSN1S1), αS2-casein (CSN1S2), β-casein (CSN2), and κ-casein (CSN3) [1]. αS1-Casein maintains the casein stability by binding to αS2-casein [2]. Bioactive peptides derived from β-casein contribute to the health of human cardiovascular, bone and immune system [3]. κ-Casein forms complexes with calcium ions, which enhances the nutritional value of dairy products [4]. These four types of casein furnish humans with high-quality protein sources. Therefore, to improve casein content in goat milk, it is necessary to analyze the molecular mechanism of casein synthesis in goat mammary epithelial cells (GMECs).

The synthesis of casein is a complex procedure that encompasses multiple signaling pathways and transcription factors in mammals. Janus kinase 2/signal transducer and activator of transcription 5 (JAK2/STAT5) and mechanistic target of rapamycin (mTOR) signaling pathways are crucial regulatory routes for governing the synthesis of casein [5]. After binding to membrane receptors of mammary epithelial cells, hormones such as prolactin activate JAK2 and catalyze downstream STAT5, then phosphorylated STAT5 activates the transcription of casein genes [6]. Amino acids activate mTOR pathway and initiate translation of casein genes [7]. E74-like factor 5 (ELF5), a member of the epithelial development-specific transcription factor, is associated with the regulation of casein synthesis [8]. The conserved deoxyribonucleic acid (DNA)-binding domain of ELF5 recognizes the promoter or enhancer regions to regulate target gene expression [9]. ELF5 participates in mammary gland development, placenta formation, maintenance of epithelial cell function, and milk protein synthesis [10].

To enhance the casein content in goat milk, it is indispensable to comprehend the regulatory function of ELF5 in casein synthesis. Previous studies showed that ELF5 facilitates the mammary gland development and milk protein content, it remains unclear whether ELF5 could regulate casein synthesis via JAK2/STAT5 signaling pathway. Our results furnish the molecular mechanism by which ELF5 regulates casein synthesis in goats, which offers a novel approach for enhancing the protein quality of ruminant milk.

## MATERIALS AND METHODS

### Cell culture

GMECs were isolated from three healthy Wanlin white goats at peak lactation period (2 years old, average of about 52 kg, second parturition, 45 d postpartum). The protocol for purification and cultured procedure as previously described [11]. Briefly, mammary tissue was collected using a surgical approach and washed with D-Hank’s solution. Tissue was then minced into about 1 mm^3^ cubes, plated in 60 mm culture dishes and cultured in 5% CO_2_ at 37°C. The medium was replaced every 2 days until the epithelial cells digested from the tissue block with 0.25% trypsin- ethylene diamine tetraacetic acid solution. Subsequently, fibroblasts were eliminated by differential adherence method, which adhered to culture dishes faster than GMECs. Cells were transferred to new culture dishes and purified 5 passages for subsequent experiments. The complete medium for GMECs was incorporated with 10% fetal bovine serum (11011-8615; Every Green) and 90% Dulbecco’s modified eagle medium/Ham’s F-12 (SH30023; Hyclone) medium with 10 μg/L epidermal growth factor (PHG0311; Invitrogen), 100 kU/L penicillin/streptomycin (080092569; Harbin Pharmaceutical), 5 mg/L bovine insulin (16634; Sigma-Aldrich), and 1 mg/L hydrocortisone (H0888; Sigma-Aldrich).

### Identification of mammary epithelial cells

To determine the purity GMECs, immunofluorescence and oil red O staining were performed. Cells were washed with phosphate buffer solution, fixed with 4% paraformaldehyde, permeabilized with Triton, and blocked with 5% bovine serum albumin at room temperature for 1 h. Subsequently, the cells were incubated with Cytokeratin 18 (66187; Proteintech; 1:800) and β-casein antibody (bs-0466R; Bioss; 1:500) for 1 h in the dark. Thereafter, the cells were incubated with Alexa Fluor 488-conjugated goat anti-mouse immunoglobulin G (IgG) (CW0102; CWBIO; 1:5,000). To ascertain whether GMECs possess normal secretory function, cells were stained with the oil red O staining kit (G1262; Solarbio) and observed through a microscope.

### Construction of overexpression vector and small interfering ribonucleic acid

Based on goat *ELF5* mRNA sequence (GenBank accession no. XM_005690080.3), the forward primer and reverse primer are CGCGGATCCCTCACGGTGATGTTGGAC and GATGGAGCAGATCATAGCT, respectively. Employing cDNA samples derived from the mammary gland of goats at peak lactation. The coding sequence of the goat *ELF5* gene was cloned into the BamHI/EcoRI sites of pcDNA3.1 using T4 DNA ligase (Takara), generating the pcDNA3.1-ELF5 expression vector. By utilizing Invitrogen online software, specific small interfering ribonucleic acid (siRNA) targeting goat *ELF5* gene was designed, concurrently with an irrelevant negative control (NC) sequence siRNA-NC (Sangon, China). The sequence of siRNA-ELF5 is as follows: sense: GCAGAGCUCUGAGGUACUATT, antisense: UAGUACC UCAGAGCUCUGCTT. The sequence of siRNA-NC is: sense: UUCUCCGAACGUGUCACGUT, antisense: ACG UGACACGUUCGGAGAATT.

### Cell transfection

For *ELF5* gene overexpression assay, pcDNA3.1-NC or pcDNA3.1-ELF5 was transfected into GMECs by Lipofectamine 3000 (Invitrogen). For the knockdown of *ELF5* gene, siRNA-NC or siRNA-ELF5 (100 nM) was transfected into cells by Lipofectamine RNAiMAX (Invitrogen). For the inhibition of JAK2/STAT5 pathway, JAK2 inhibitor AG490 (30 μM, MedChemExpress) or STAT5 inhibitor STAT5-IN-1 (50 μM, MedChemExpress) were treated with cells. For rescue experiment with STAT5 inhibitor and ELF5 overexpression, cells seeded in 6-well plates were grown to 80% confluence. After pre-treatment with STAT5-IN-1 (50 μM) for 1 h, transfection was performed with pcDNA3.1-NC or pcDNA3.1-ELF5 using Lipofectamine 3,000 according to the manufacturer’s protocol. Total protein was collected 48 h after transfection for Western blot analysis. Similarly, after pre-treatment with STAT5-IN-1 (50 μM) for 1 h, transfection was performed with siRNA-NC or siRNA-ELF5 (100 nM) using Lipofectamine RNAiMAX for 48 h. For rescue experiment with JAK2 inhibitor and ELF5 overexpression, cells seeded in 6-well plates were grown to 80% confluence. After pre-treatment with AG490 (30 μM) for 1 h, transfection was performed with pcDNA3.1-NC or pcDNA3.1-ELF5 using Lipofectamine 3,000 for 48 h. Similarly, after pre-treatment with AG490 (30 μM) for 1 h, transfection was performed with siRNA-NC or siRNA-ELF5 (100 nM) using Lipofectamine RNAiMAX for 48 h. Besides, for the interaction assay of ELF5 and STAT5, cells were transfected with pCMV-STAT5a or pCMV-NC. After 48 h treatment, cells were harvested to extract total RNA and protein.

### Cell viability and apoptosis assays

Cell viability was carried out by cell counting kit-8 (CCK-8) assay in GMECs. After *ELF5* gene overexpression or interference for 24, 48, and 72 h, the cells were incubated with CCK-8 reagent (5 g/L, CA1210; Solarbio) at 37°C for 2 h. A microplate reader (TECAN Spark) was employed to measure the absorbance of cell culture medium at 450 nm. Then, cell apoptosis was carried out by flow cytometry. The cells were transfected with pcDNA3.1-ELF5 or si-ELF5 for 48 h, subsequently cell apoptosis was determined by using the Annexin V-FITC/PI dual staining cell apoptosis detection kit (KGA1102-100; KeyGen) by a flow cytometer (BD Accuri C6puls).

### RNA extraction and quantitative real-time polymerase chain reaction

Total RNA of GMECs was extracted by SPARKeasy (AC0-202-B; Sparkjade). The quality of RNA was evaluated by employing a NanoDrop One spectrophotometer (Thermo Fisher Scientific), where the ratio of absorbance at 260 nm to 280 nm ranged from 1.8 to 2.1. The integrity of RNA was determined through the analysis of 28S and 18S rRNA via agarose gel electrophoresis, with a ratio of 28S/18S approximately 2:1. For the quantitative analysis of cell proliferation and apoptosis marker genes as well as casein genes, the first strand cDNA was generated by utilizing purified total RNA through the PrimeScriptRT reagent kit (PC7001; Aidlab), which incorporates a gDNA eraser. The synergetic binding reagent green kit (PC3301; Aidlab) was used for quantitative real-time polymerase chain reaction (qPCR). Employing ubiquitous expression transcript (*UXT*) as an internal control gene, the qPCR primer sequences are presented in Table 1. All qPCR reactions were conducted on the CFX96 sequence detection system (Bio-Rad), and the data were analyzed by relative quantification (2^−ΔΔCt^) method.

### Western blot

Total protein of GMECs was harvested after treated with radio immunoprecipitation assay lysis buffer (R0010; Solarbio) containing protease inhibitors (G2006-250UL; Servicebio). The protein assay kit (P0010S; Beyotime) was employed to detect protein concentration. Proteins were separated via sodium dodecyl sulfate-polyacrylamide gel electrophoresis and transferred onto polyvinylidene fluoride membrane (IPVHO0005; Merck) by BG-transBLOT. The membrane was blocked with 5% skim milk for 2 h (D8340; Solarbio) and subjected to incubation with the primary antibody at 4°C overnight. Then the membrane incubated with the secondary antibody. Primary antibodies contains ELF5 (sc-166653, SantaCruz; 1:1,000), αS2-casein (bs-10034R, Bioss; 1:2,000), κ-casein (bs-10031R; Bioss; 1:1,000), αS1-casein (bs-10033R; Bioss; 1:1,000), β-casein (sc-166530; SantaCruz; 1:1,000), p-JAK2 (ab32101; Abcam; 1:1,000), p-STAT5 (9351S; 1:1,000), JAK2 (3230; CST; 1:1,000), STAT5 (610191; BD; 1:1,000), proliferating cell nuclear antigen (PCNA, 10205-2-APl Proteintech, 1:5,000), cyclin dependent kinase 2 (CDK2, 10122-1-AP; Proteintech, 1:10,000), B-cell lymphoma-2-associated X (BAX, 50599-2-lg; Proteintech, 1:10,000), Caspase3 (19677-1-AP; Proteintech, 1:2,000), β-Tubulin (CW0098M; CWBIO, 1:5,000). Secondary antibody contains horseradish peroxidase-conjugated goat anti-rabbit-IgG (CW0102; CWBIO; 1:5,000) and goat anti-mouse-IgG (CW0103; CWBIO; 1:5,000). Signals were measured using an enhanced chemiluminescent Western blot system (AllianceQ9Advanced). The intensity of indicated bands was quantified by densitometry using ImageJ software (http://imagej.nih.gov/ij/). The relative abundance of proteins is normalized to β-Tubulin. The relative abundance of p-JAK2 and p-STAT5 is normalized to total JAK2 or STAT5.

### Co-immunoprecipitation assay

Following 48 h co-transfection with pcDNA3.1-ELF5-MYC and PCMV-STAT5-3xFLAG vectors, GMECs were lysed for 20 min by immunoprecipitation lysis buffer. Subsequently, the mixture was centrifuged at 13,000×g for 10 min, and the supernatant was taken as the total protein. The Protein G beads were washed with pre-cooled phosphate buffer solution and diluted to a 50% suspension. Subsequently, Protein G beads (L-1202A; Biolinkedin) pre-washing was employed to eliminate non-specific adsorption from the cell lysate. Then centrifuged total cell protein and Protein G beads suspension were mixed and incubated at 4°C for 1 h. Centrifugation was performed at 600×g for 3 min, and the supernatant was collected. The antibody and Protein G beads were pre-incubated. The diluted solution was added Mouse-MYC-tag antibody (60003-2-Ig; Proteintech), Mouse-FLAG-tag antibody (66008-4-Ig; Proteintech) and Protein G beads suspension. The mixtures were incubated at 4°C for 1 h and centrifuged at 600×g for 3 min to retain the precipitates for three times. Subsequently, the samples were collected for Western blot detection.

### Statistical analysis

The data were conducted by SPSS 22.0 (IBM) and presented as mean±standard error of the mean (SEM) of three biological replicates. When only two groups were compared, the data were analyzed through the t-test (two-tailed). For multiple comparisons, a general linear model program was utilized for ANOVA with Tukey’s test. A difference was regarded as statistically significant (* p<0.05, ** p<0.01).

## RESULTS

### Isolation and identification of goat mammary epithelial cells

**Figure 1:**
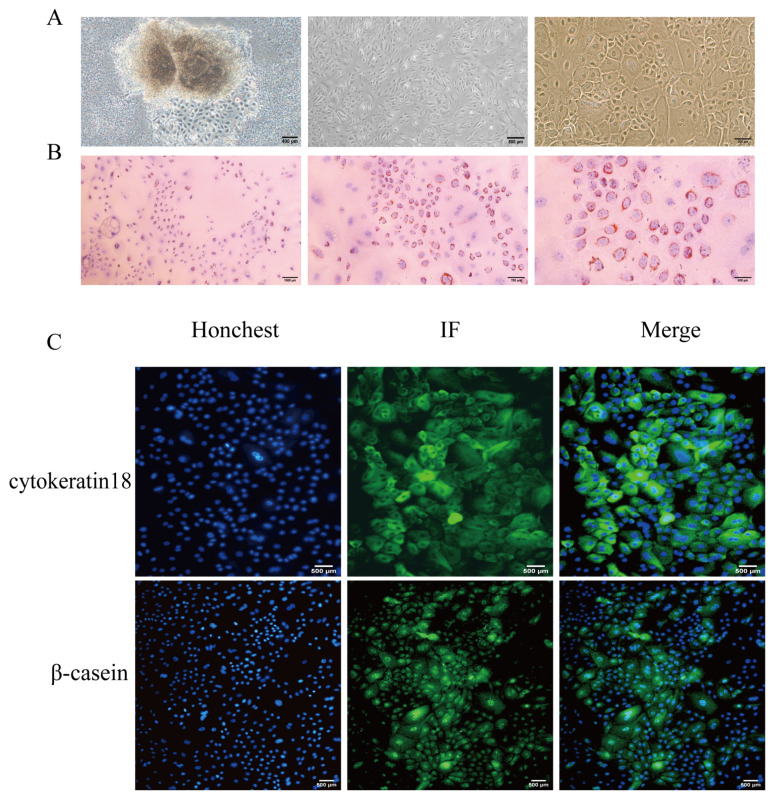
Isolation, cultivation, and identification of GMECs. (A) Morphological observation of adherent cells. Mammary epithelial cells on day 7 of isolation (left panel, scale bar = 400 μm); mammary epithelial cells after 5 passages of purification (middle panel, scale bar = 800 μm); mammary epithelial cells after 5 passages of purification (right panel, scale bar = 200 μm). (B) Cell identification by Oil Red O Staining. Scale bars correspond to 1,000 μm (left panel), 700 μm (middle panel), 400 μm (right panel). (C) Immunofluorescence detection of cytokeratin 18 and β-casein (scale bar = 500 μm). GMECs, goat mammary epithelial cells.

Primary GMECs were migrating out from the goat mammary tissue, and purified cells were attained after 5 passages of purified culture (Figure 1A). Oil red O staining showed that numerous lipid droplet milk globules were dispersed around the GMECs, signifying that cells possess normal secretory function (Figure 1B). Immunofluorescence assays showed mammary epithelial cell markers cytokeratin 18 and β-casein were positive staining (Figure 1C).

### Overexpression and interference of *ELF5* gene

**Figure 2:**
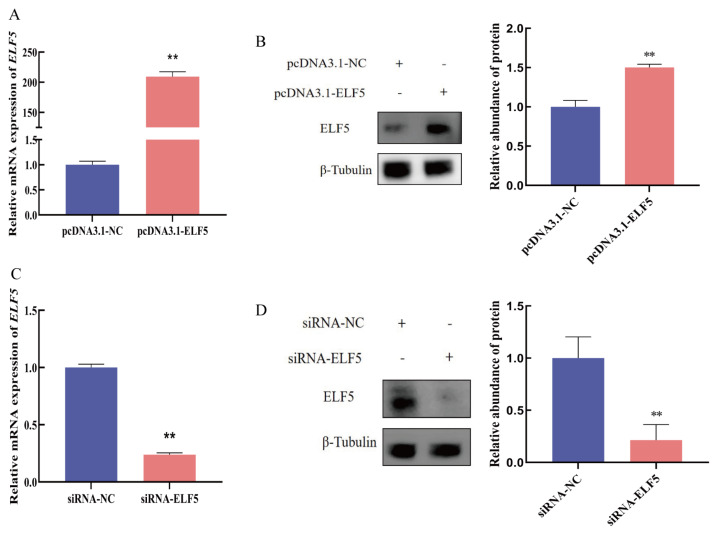
Construction of *ELF5* gene overexpression vector and siRNA. (A, B) The mRNA and protein expression of ELF5 in cells transfected with pcDNA3.1-ELF5 or pcDNA3.1-NC for 48 h. (C, D) The mRNA and protein expression of ELF5 in cells transfected with siRNA-ELF5 or siRNA-NC (100 nM) for 48 h. The relative protein abundance was normalized to β-tubulin. Values are presented as mean±SEM. ** p<0.01. siRNA, small interfering ribonucleic acid; NC, negative control; SEM, standard error of the mean.

To explore the regulatory mechanism of ELF5 on casein synthesis, overexpression vector and siRNA of *ELF5* gene were performed. qPCR and Western blot results indicated that pcDNA3.1-ELF5 vector could significantly enhance the mRNA and protein expression of ELF5 compared with the pcDNA3.1-NC group (p<0.001, Figures 2A, 2B, Supplement 1). Similarly, compared with the siRNA-NC group, siRNA-ELF5 markedly decreased the mRNA (p<0.001) and protein expression (p = 0.005) of ELF5 (Figures 2C, 2D, Supplement 2).

### Cell proliferation and apoptosis after *ELF5* gene overexpression

**Figure 3:**
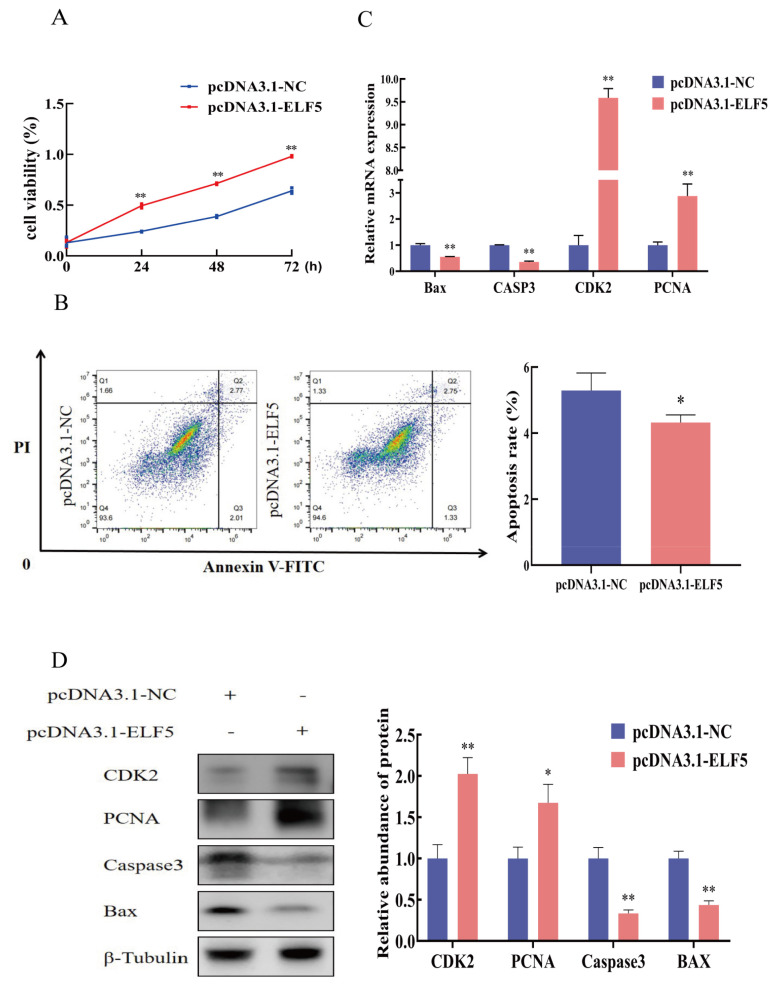
*ELF5* gene overexpression regulates proliferation and apoptosis of GMECs. (A) Cell viability was measured after pcDNA3.1-ELF5 or pcDNA3.1-NC transfection using CCK-8 assay kit. (B) Cell apoptosis was determined by flow cytometry after pcDNA3.1-ELF5 or pcDNA3.1-NC transfection for 48 h. The (C) mRNA and (D) protein expression of CDK2, PCNA, Caspase3, and Bax after transfected with pcDNA3.1-ELF5 or pcDNA3.1-NC for 48 h. The relative protein abundance was normalized to β-tubulin. Values are presented as mean±SEM. * p<0.05, ** p<0.01. NC, negative control; GMECs, goat mammary epithelial cells; SEM, standard error of the mean.

The CCK-8 results showed that cell viability was significantly enhanced after *ELF5* gene overexpression for 24, 48, and 72 h (p<0.01, Figure 3A). Flow cytometry assay revealed cell apoptosis rate was decreased by overexpressing *ELF5* gene (p = 0.043, Figure 3B). The results of qPCR and Western blot showed that *ELF5* gene overexpression markedly increased the mRNA and protein expression of proliferation marker genes PCNA and CDK2, and conspicuously decreased the expression of apoptosis marker genes Bax and Caspase3 (p< 0.05, Figures 3C, 3D, Supplement 3).

### Cell proliferation and apoptosis after *ELF5* gene interference

**Figure 4:**
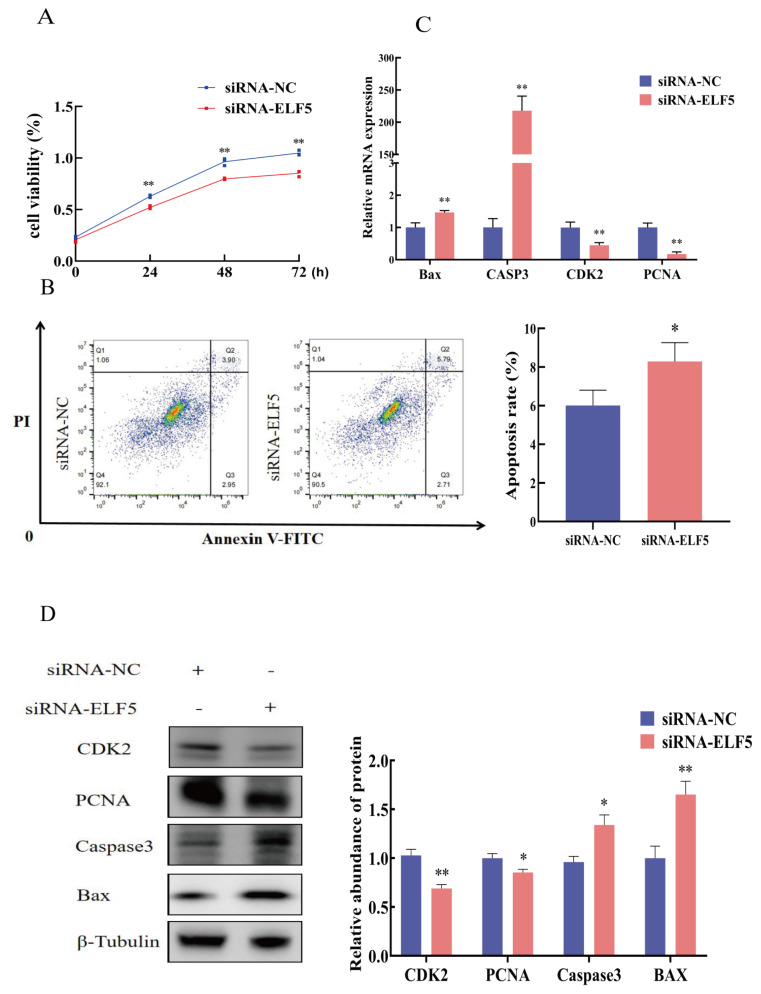
*ELF5* gene interference regulates proliferation and apoptosis of GMECs. (A) Cell viability was measured after siRNA-ELF5 or siRNA-NC (100 nM) transfection using CCK-8 assay kit. (B) Cell apoptosis was determined by flow cytometry after siRNA-ELF5 or siRNA-NC (100 nM) transfection for 48 h. The (C) mRNA and (D) protein expression of CDK2, PCNA, Caspase3, and Bax after transfected with siRNA-ELF5 or siRNA-NC (100 nM) for 48 h. The relative protein abundance was normalized to β-tubulin. Values are presented as mean±SEM. * p<0.05, ** p<0.01. siRNA, small interfering ribonucleic acid; NC, negative control; GMECs, goat mammary epithelial cells; SEM, standard error of the mean.

The CCK-8 results showed that cell viability was significantly decreased after *ELF5* gene interference for 24, 48, and 72 h (p<0.01, Figure 4A). Flow cytometry assay revealed cell apoptosis rate was increased by knocking down *ELF5* gene (p = 0.035, Figure 4B). qPCR and Western blot results showed that *ELF5* gene interference markedly decreased the mRNA and protein expression of PCNA and CDK2, and increased the expression of Bax and Caspase3 (p<0.05, Figures 4C, 4D, Supplement 4).

### Casein synthesis by *ELF5* gene overexpression and interference

**Figure 5:**
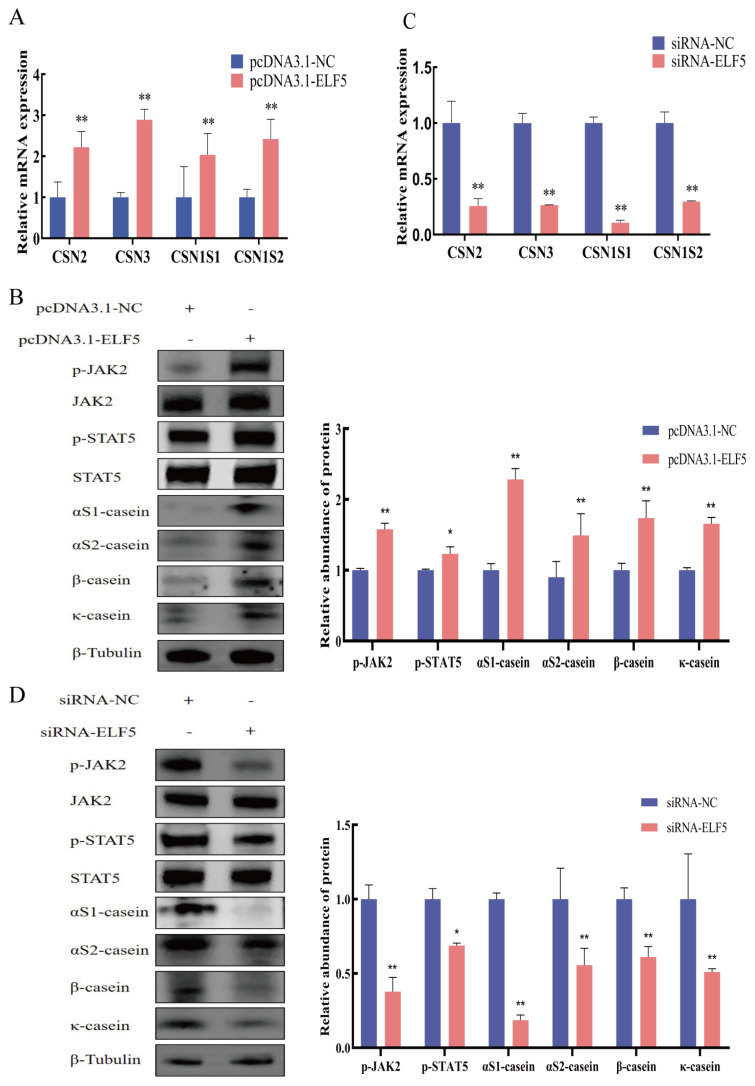
ELF5 regulate casein synthesis in GMECs. (A, B) The expression of αS1-casein, αS2-casein, β-casein, κ-casein, phosphorylated JAK2 and STAT5 after transfection with pcDNA3.1-ELF5 or pcDNA3.1-NC for 48 h. (C, D) The expression of αS1-casein, αS2-casein, β-casein, κ-casein, p-JAK2, and p-STAT5 after transfection with siRNA-ELF5 or siRNA-NC (100 nM) for 48 h. The relative protein abundance of αS1-casein, αS2-casein, β-casein, and κ-casein was normalized to β-tubulin. The relative protein abundance of p-JAK2 and p-STAT5 was normalized to total JAK2 and STAT5, respectively. Values are presented as mean±SEM. * p<0.05, ** p<0.01. siRNA, small interfering ribonucleic acid; NC, negative control; GMECs, goat mammary epithelial cells; SEM, standard error of the mean.

In comparison with control group, *ELF5* gene overexpression conspicuously elevated the mRNA and protein expression of αS1-casein, αS2-casein, β-casein, and κ-casein as well as the activity of phosphorylated JAK2 and STAT5 (p<0.05, Figures 5A, 5B, Supplement 5). In addition, *ELF5* gene interference significantly decreased the expression of αS1-, αS2-, β-, and κ-casein, and reduced the activity of JAK2/STAT5 signaling pathway (p<0.05, Figures 5C, 5D; Supplement 6).

### Casein synthesis by ELF5 mediated JAK2/STAT5 signaling pathway

**Figure 6:**
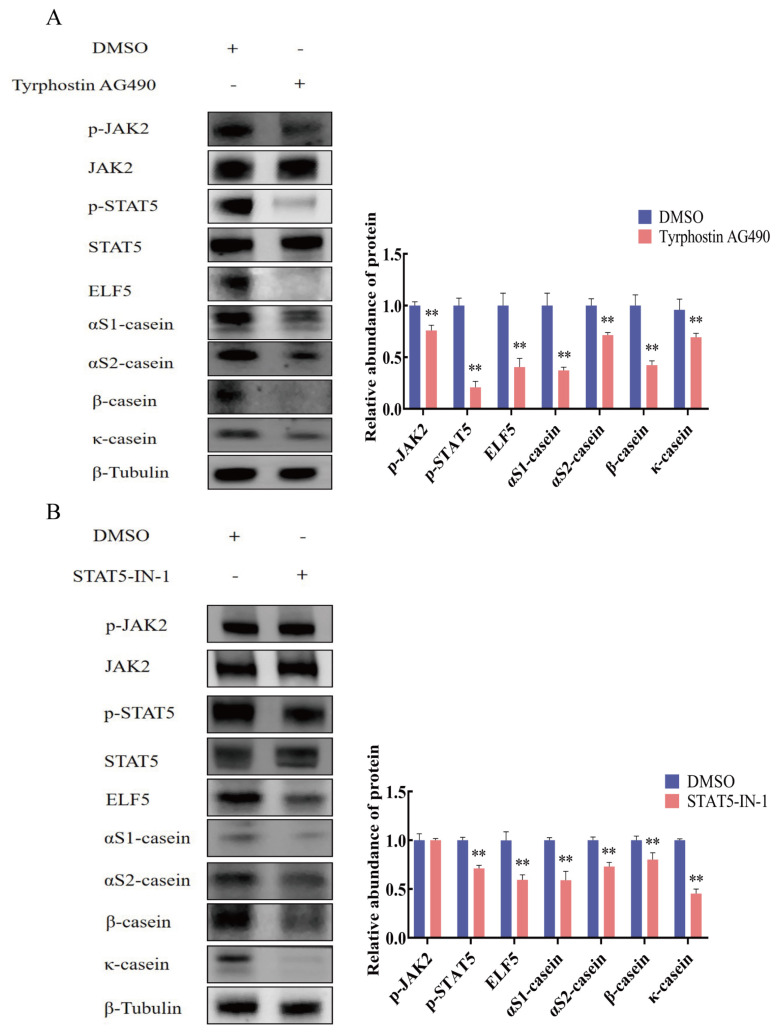
The protein abundance of caseins after JAK2 and STAT5 inhibition in GMECs. (A) The expression of αS1-casein, αS2-casein, β-casein, κ-casein, and phosphorylated JAK2 and STAT5 after JAK2 inhibitor Tyrphosting AG490 (30 μM) treatment for 48 h. (B) The expression of αS1-casein, αS2-casein, β-casein, κ-casein, and p-STAT5 after STAT5 inhibitor STAT5-IN-1 (50 μM) treatment for 48 h. The relative protein abundance of αS1-casein, αS2-casein, β-casein, and κ-casein was normalized to β-tubulin. The relative protein abundance of p-JAK2 and p-STAT5 was normalized to total JAK2 and STAT5, respectively. Values are presented as mean±SEM. ** p<0.01. GMECs, goat mammary epithelial cells; SEM, standard error of the mean.

Regulation of casein synthesis by JAK2/STAT5 signaling pathway was confirmed by Western blot assay. The protein abundance of p-JAK2, p-STAT5, αS1-casein, αS2-casein, β-casein, and κ-casein were reduced after JAK2 inhibitor AG490 treatment (p<0.01, Figure 6A; Supplement 7). Then, the protein abundance of p-STAT5 and four caseins were significantly reduced in STAT5 inhibitor STAT5-IN-1 group (p<0.01, Figure 6B, Supplement 8).

**Figure 7:**
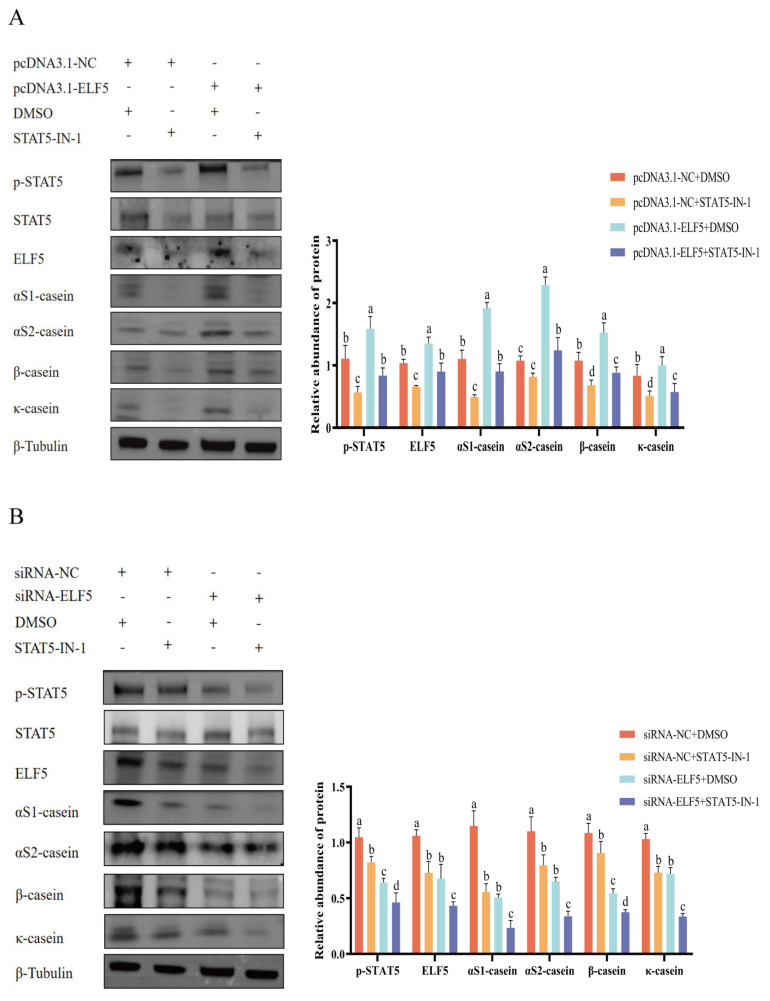
ELF5 mediates casein synthesis by STAT5 activity in GMECs. (A) Cells were treated with STAT5-IN-1 (50 μM) or DMSO, followed by pcDNA3.1-ELF5 or pcDNA3.1-NC transfection for 48 h. (B) Cells were treated with STAT5-IN-1 (50 μM) or DMSO, followed by siRNA-ELF5 or siRNA-NC (100 nM) transfection for 48 h. The protein abundances of αS1-casein, αS2-casein, β-casein, κ-casein, and p-STAT5 were detected. The relative protein abundance of αS1-casein, αS2-casein, β-casein, and κ-casein was normalized to β-tubulin. The relative protein abundance of p-STAT5 was normalized to total STAT5. Values are presented as mean±SEM. ^a–d^ Different lowercase letters represent significant differences (* p<0.05). siRNA, small interfering ribonucleic acid; NC, negative control; GMECs, goat mammary epithelial cells; SEM, standard error of the mean.

**Figure 8:**
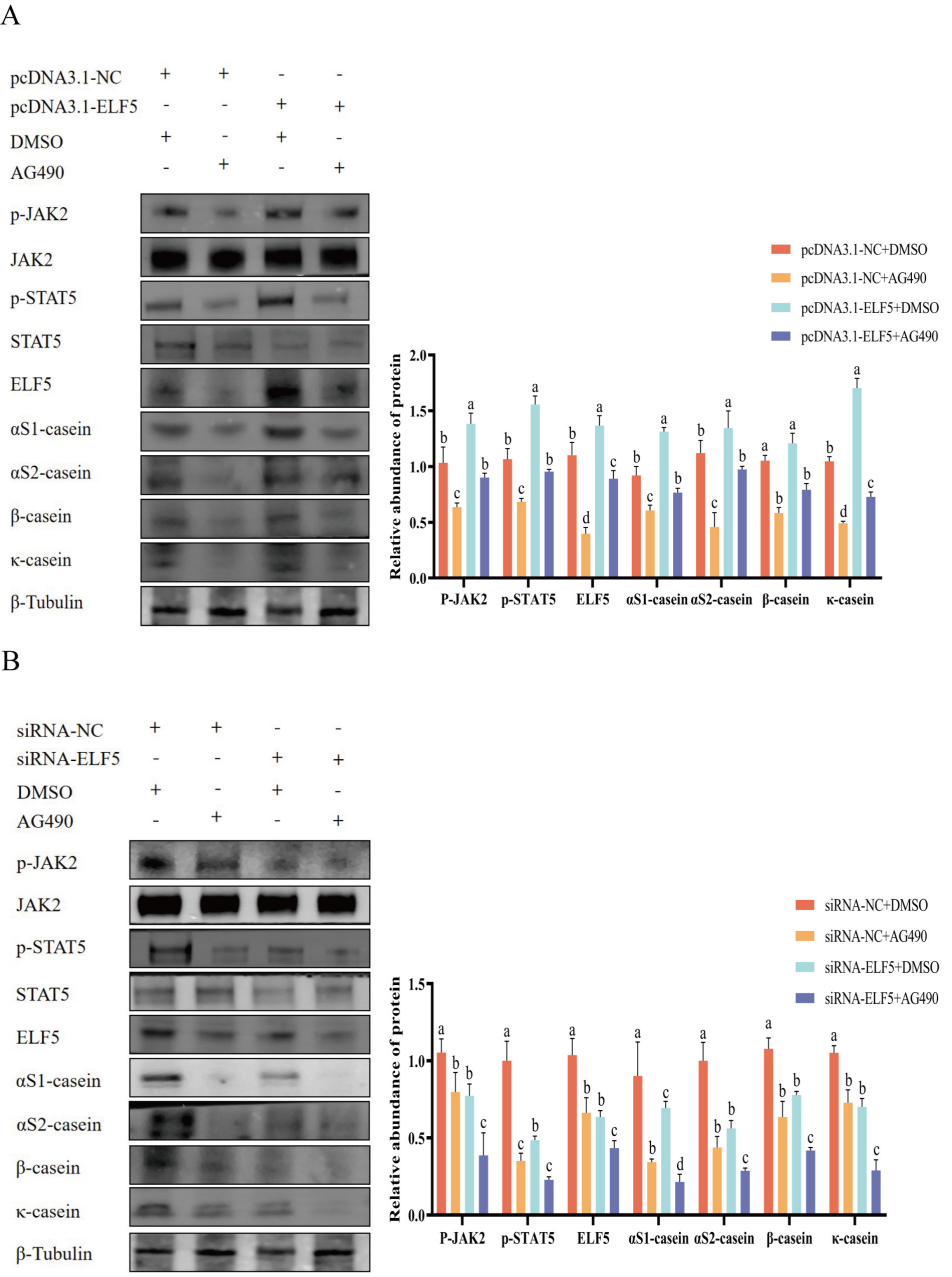
ELF5 mediates casein synthesis by JAK2 and STAT5 activity in GMECs. (A) Cells were treated with Tyrphosting AG490 (30 μM) or DMSO, followed by pcDNA3.1-ELF5 or pcDNA3.1-NC transfection for 48 h. (B) Cells were treated with Tyrphosting AG490 (30 μM) or DMSO, followed by siRNA-ELF5 or siRNA-NC (100 nM) transfection for 48 h. The protein abundances of αS1-casein, αS2-casein, β-casein, κ-casein, p-JAK2, and p-STAT5 were detected. The relative protein abundance of αS1-casein, αS2-casein, β-casein, and κ-casein was normalized to β-tubulin. The relative protein abundance of p-JAK2 and p-STAT5 was normalized to total JAK2 and STAT5, respectively. Values are presented as mean±SEM. ^a–d^ Different lowercase letters represent significant differences (* p<0.05). NC, negative control; siRNA, small interfering ribonucleic acid; GMECs, goat mammary epithelial cells; SEM, standard error of the mean.

Moreover, to validate whether ELF5 promotes casein synthesis via JAK2/STAT5 signaling pathway, the rescue experiments were carried out. Overexpression of *ELF5* gene upregulated p-STAT5 activity and casein expression, while co-treatment with STAT5 inhibitor abrogated the increase of p-STAT5 activity and casein expression (Figure 7A, Supplement 9). Simultaneously, knockdown of *ELF5* gene downregulated p-STAT5 activity and casein expression, whereas co-treatment with STAT5 inhibitor further reduced activity of p-STAT5 and abundance of caseins (Figure 7B, Supplement 10). Furthermore, co-treatment with *ELF5* gene overexpression and JAK2 inhibitor abrogated the increase of JAK2/STAT5 activity and casein expression (Figure 8A, Supplement 11). Besides, co-treatment with *ELF5* gene interference and JAK2 inhibitor further decreased JAK2/STAT5 activity and casein expression (Figure 8B, Supplement 12).

### Immunoprecipitation of ELF5 and STAT5

**Figure 9:**
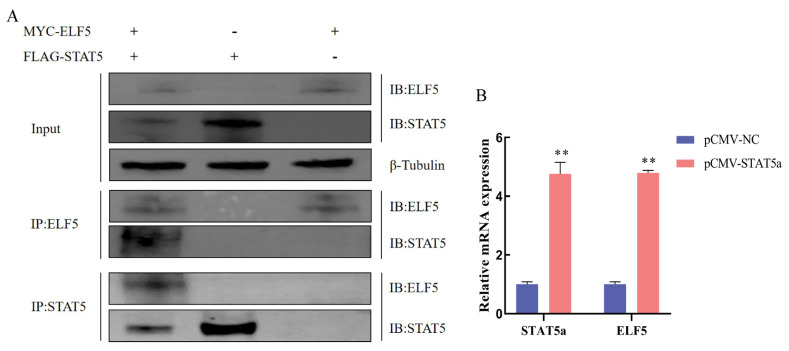
The interaction between ELF5 and STAT5 in GMECs. (A) Cells were transfected with ELF5-MYC and STAT5-FLAG for 48 h. Then, total protein of cells was extracted and incubated with MYC (or FLAG) tag antibody and protein G magnetic beads at 4°C overnight. The protein abundance of ELF5 and STAT5 was detected. (B) The mRNA expression of *STAT5a* and *ELF5* gene was measured after pCMV-STAT5a and pCMV-NC transfection for 48 h. Values are presented as mean±SEM. ** p<0.01. GMECs, goat mammary epithelial cells; NC, negative control; SEM, standard error of the mean.

**Figure 10:**
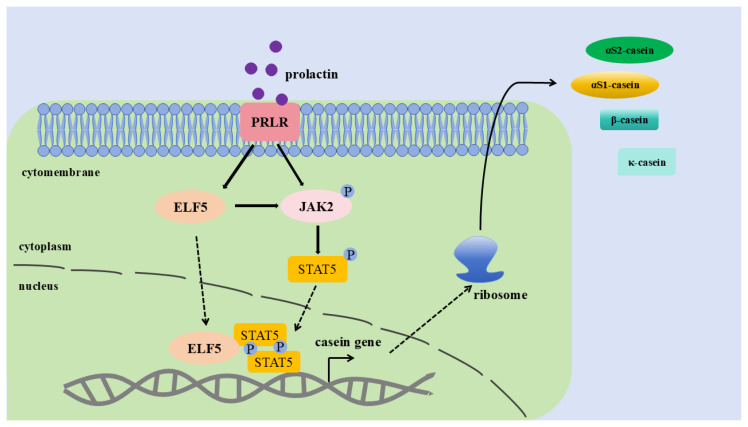
Molecular mechanism of ELF5 promotes casein synthesis by enhancing the activity of JAK2/STAT5 signaling pathway in goat mammary epithelial cells. PRLR stands for prolactin receptor.

To verify whether ELF5 interacts with STAT5, an immunoprecipitation assay was carried out. We demonstrated that ELF5 interacted with STAT5 in GMECs by Western blot assay (Figure 9A). Moreover, qPCR result showed that the mRNA level of ELF5 was highly elevated after *STAT5a* gene overexpression (p<0.001, Figure 9B, Supplement 13). These results further validate that ELF5 could regulate casein synthesis via JAK2/STAT5 signaling pathway by interacting with STAT5 (Figure 10).

## DISCUSSION

ELF5 plays crucial roles in cell proliferation, differentiation, development, cell apoptosis and milk synthesis of mammary gland [12]. In human mammary epithelial cell lines, prolactin upregulated the expression of ELF5, and milk protein synthesis was blocked in the absence of prolactin, while overexpression of ELF5 restored milk protein synthesis [13]. In the *ELF5* gene knocked out mouse model, there was a notable stagnation of mammary development, encompassing a decrease in milk protein synthesis [14]. *ELF5* gene overexpression could increase the milk protein content in an inducible transgenic model [15]. In lactating dairy goats, ELF5 binds to the lactalbumin gene promoter sequence to promote the synthesis of α-lactalbumin and β-lactoglobulin [16]. In our previous study, *ELF5* and *CSN2* gene exhibit higher expression levels during the peak and mid-lactation periods compared to dry lactation period of goats [17]. Our study indicated that ELF5 promotes the synthesis of caseins in GMECs by overexpression and interference of *ELF5* gene. We investigated the mechanism of ELF5 on casein synthesis and refined the function of ELF5 in mammary lactation of goats.

During pregnancy, ELF5 regulates the lactation process in mammary gland, facilitating the differentiation of mammary stem cells into secretory cell types [18]. Furthermore, recombinant ELF5 activates the promoter of whey proteins, facilitating the development of mouse mammary epithelial cells [19]. Moreover, ELF5 regulates the proliferation and apoptosis of epithelial cells in the respiratory system, such as secretory cells and alveolar type II cells [20]. In breast cancer cells, ELF5 binds to CD24 gene promoter region, thereby regulating CD24 expression to inhibit cell proliferation and invasion [21,22]. In the present study, in addition to enhancing casein synthesis, ELF5 also promotes cell proliferation and inhibits cell apoptosis in GMECs. which predicts a tight link between casein synthesis and cell activity.

In mammals, endocrine hormones such as prolactin, insulin, and hydrocortisone maintain the lactation process and regulate milk protein synthesis through JAK2/STAT5 and mTOR pathways [23]. Phosphorylated STAT5 translocates into the nucleus, and binds to gamma-interferon activation sequence, thereby facilitating the transcription of casein gene promoter. JAK2/STAT5 regulate the expression of suppressor of cytokine signaling 3 and sterol regulatory element-binding protein implicated in casein synthesis [24,25]. Furthermore, phosphorylated STAT5 activates mTOR signaling pathway to facilitate the translation of casein genes [26]. In dairy cow mammary epithelial cells, STAT5 regulates the expression of amino acid transporters and promotes milk protein synthesis, and JAK2/STAT5 and mTOR pathways compensate to maintain milk protein synthesis [27]. In our study, inhibition of JAK2 or STAT5 activity decreased casein expression of goats, confirming the function of JAK2/STAT5 in milk protein synthesis. Furthermore, the overexpression and knockdown of *ELF5* gene and the inhibition of JAK2/STAT5 signaling pathway were employed, which demonstrated that ELF5 mediates the regulation of casein synthesis via JAK2/STAT5 signaling pathway.

The expression of ELF5 in mammary epithelial cells is regulated by STAT5, while ELF5 concurrently enhanced STAT5 activity. The specific knockout of ELF5 in the mouse mammary gland reduced the phosphorylation activity of STAT5, and inhibited the synthesis and secretion of milk protein, while overexpression of *ELF5* gene significantly promoted the production of milk protein [28]. Compared with the dry lactation stage of yaks, ELF5 was highly expressed in the lactation stage, which enhanced the phosphorylation activity of STAT5 and affected the expression of key genes in milk protein synthesis [29]. ELF5 binds to the *STAT5* gene promoter sequence in the mammary tissue of dairy cows, enhance the transcription level and protein activity of STAT5, and regulate milk protein synthesis [30]. ELF5 and STAT5 modulate the differentiation of mammary epithelial cells to jointly govern the synthesis of milk proteins [31]. STAT5 could interact with ELF5 to augment the transcriptional activity of casein genes [32]. The co-immunoprecipitation experiment substantiates the interaction between ELF5 and STAT5, and the phosphorylation of STAT5 is up-regulated due to *ELF5* gene overexpression, further intensifying the activation of JAK2/STAT5 pathway to facilitate milk production.

In dairy cow mammary epithelial cells, the proliferation, apoptosis, and casein synthesis were regulated by nutrition and hormone mediated JAK2/STAT5 and mTOR signaling pathway [33,34]. JAK2/STAT5 pathway promotes the expression of cell cycle-related genes cyclin D1 and cyclin E1, inhibiting the expression of cell cycle inhibitory factors p21 and p27, and apoptotic signaling pathway Caspase3 and Caspase8, thereby suppressing cell apoptosis and facilitating cell proliferation [20]. The vitality and quantity of mammary epithelial cells constitute the key indicator influencing milk protein secretion, and high vitality cells could effectively utilize nutrients for the synthesis of milk proteins [35]. In this study, ELF5 elevated the expression of cell proliferation markers CDK2 and PCNA, whilst inhibiting the expression of cell apoptosis markers Bax and Caspase3. Cell survival influences cellular metabolic activity, casein synthesis capacity, and the maintenance of lactation function; high cell survival facilitates casein synthesis in mammary epithelial cells [36–38]. While, the mechanism by which ELF5 mediates JAK2/STAT5 pathway to regulate cell activity still needs to be further studied.

## CONCLUSION

ELF5 promotes cell viability and upregulated casein synthesis through JAK2/STAT5 signaling pathway in GMECs. ELF5 augments the activation of JAK2/STAT5 pathway by interacting with STAT5, thereby facilitating casein synthesis of goats. Our study provides a theoretical basis for increasing casein yield and improving milk protein quality in goats.

## Figures and Tables

**Table 1:** Primers used for qPCR of genes

National Center for Biotechnology Information accession	Gene	Primer sequence (5′−3′)	Length (bp)
XM_005690080.3	*ELF5*	F: AGCATCCGCTCACAAGGT	180
		R: AAGCAGGTCTCGCACAAA	
XM_018049127.1	*CSN1S1*	F: TCCACTAGGCACACAATACACTGA	61
		R: GCCAATGGGATTAGGGATGTC	
NM_001285585.1	*CSN1S2*	F: CTGGTTATGGTTGGACTGGAAAA	76
		R: AACATGCTGGTTGTATGAAGTAAAGTG	
XM_005681721.2	*CSN2*	F: CCCAGGCACAGTCTCTAGTCT	196
		R: GGCTCAACTGGATATTTAGGGA	
NM_001285587.1	*CSN3*	F: AGGTGCAATGATGAAGAGTTTTTTC	66
		R: CCCAAAAATGGCAGGGTTAA	
XM_005688167.3	*PCNA*	F: GCTGTTACCATAGAGATGAATG	107
		R: ATACTGAGTGTTACTGTAGGAG	
NM_001285608.1	*CDK2*	F: TTTGCTGAGATGGTGACCCG	169
		R: TAAAATCCTGCCTGGCCCAC	
XM_018062750.1	*BAX*	F: TGCTTCAGGGTTTCATCC	116
		R: CTTCAGACACTCGCTCAG	
XM_018041755.1	*CASP3*	F: TATTGAGACAGACAGTGGTT	295
		R: GAGCATAGACATGATACAAGG	
XM_018065112.1	*STAT5a*	F: CCATCGACCTGGACAATCCC	96
		R: CGACTTGGTGCTCTGCCTTCTT	
NM_001037471	*UXT*	F: CAGCTGGCCAAATACCTTCAA	125
		R: GTGTCTGGGACCACTGTGTCAA	

qPCR, quantitative real-time polymerase chain reaction.

## SUPPLEMENTARY MATERIAL

**Supplement 1**. The full Western blot image of Figure 2B.

**Supplement 2**. The full Western blot image of Figure 2D.

**Supplement 3**. The full Western blot image of Figure 3D.

**Supplement 4**. The full Western blot image of Figure 4D.

**Supplement 5**. The full Western blot image of Figure 5B.

**Supplement 6**. The full Western blot image of Figure 5D.

**Supplement 7**. The full Western blot image of Figure 6A.

**Supplement 8**. The full Western blot image of Figure 6B.

**Supplement 9**. The full Western blot image of Figure 7A.

**Supplement 10**. The full Western blot image of Figure 7B.

**Supplement 11**. The full Western blot image of Figure 8A.

**Supplement 12**. The full Western blot image of Figure 8B.

**Supplement 13**. The full Western blot image of Figure 9A.
