# Supplementary material for: ELF5 modulates casein synthesis in goat mammary epithelial cells via JAK2/STAT5 signaling pathway
Source: Anim Biosci. 2025 Oct 22;39(2):250181. doi: 10.5713/ab.25.0181 (PMC12877387; doi:10.5713/ab.25.0181)
Supplement: Supplementary file 2 [file ab-25-0181-Supplementary-2.pdf]

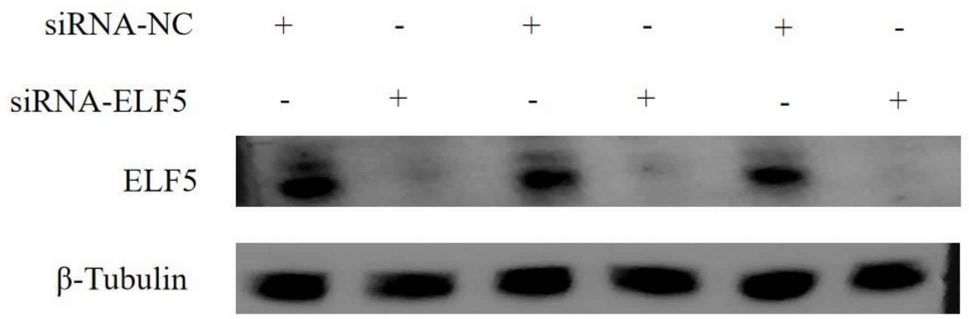

Supplement 2. The full Western blot image of Figure 2D. The protein expression of ELF5 in cells transfected with siRNA-ELF5 or siRNA-NC (100 nM) for 48 h.
