# Supplementary material for: ELF5 modulates casein synthesis in goat mammary epithelial cells via JAK2/STAT5 signaling pathway
Source: Anim Biosci. 2025 Oct 22;39(2):250181. doi: 10.5713/ab.25.0181 (PMC12877387; doi:10.5713/ab.25.0181)
Supplement: Supplementary file 3 [file ab-25-0181-Supplementary-3.pdf]

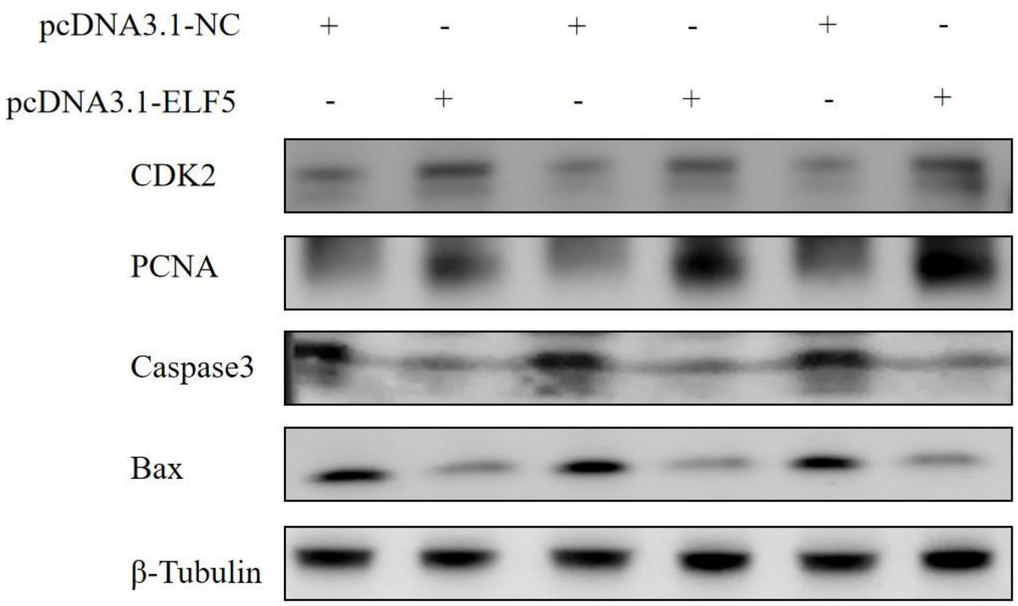

Supplement 3. The full Western blot image of Figure 3D. The protein expression of CDK2, PCNA, Caspase3, and Bax after transfected with pcDNA3.1-ELF5 or pcDNA3.1-NC for 48 h.
