# Supplementary material for: ELF5 modulates casein synthesis in goat mammary epithelial cells via JAK2/STAT5 signaling pathway
Source: Anim Biosci. 2025 Oct 22;39(2):250181. doi: 10.5713/ab.25.0181 (PMC12877387; doi:10.5713/ab.25.0181)
Supplement: Supplementary file 5 [file ab-25-0181-Supplementary-5.pdf]

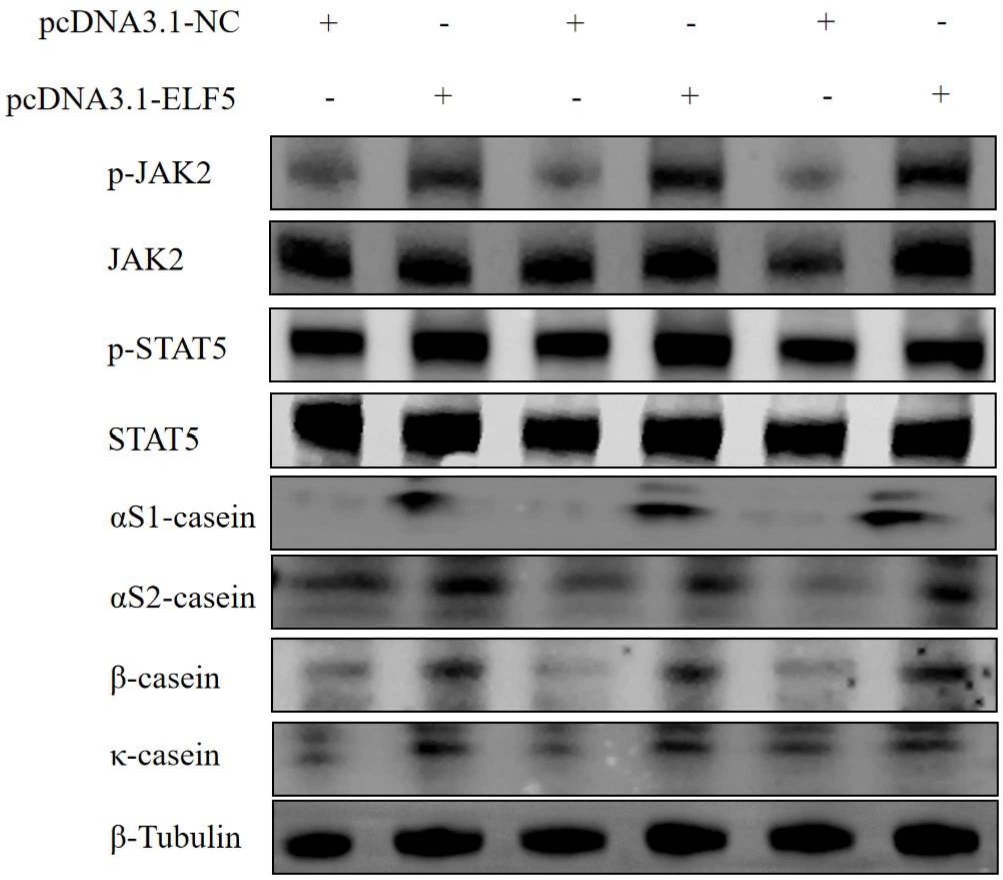

Supplement 5. The full Western blot image of Figure 5B. The expression of αS1-casein, αS2-casein, β-casein, κ-casein, phosphorylated JAK2 and STAT5 after transfection with pcDNA3.1-ELF5 or pcDNA3.1-NC for 48 h.
