# Supplementary material for: ELF5 modulates casein synthesis in goat mammary epithelial cells via JAK2/STAT5 signaling pathway
Source: Anim Biosci. 2025 Oct 22;39(2):250181. doi: 10.5713/ab.25.0181 (PMC12877387; doi:10.5713/ab.25.0181)
Supplement: Supplementary file 7 [file ab-25-0181-Supplementary-7.pdf]

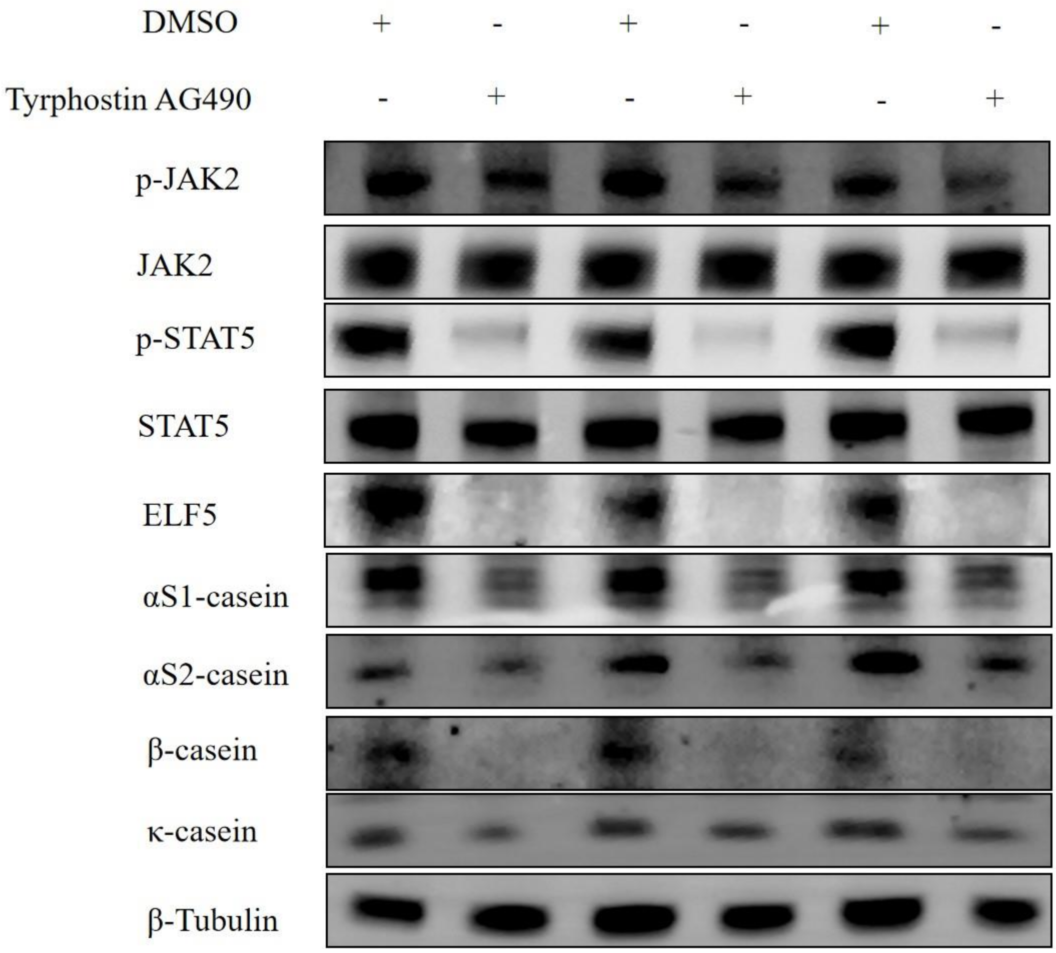

Supplement 7. The full Western blot image of Figure 6A. The expression of αS1-casein, αS2-casein, β-casein, κ-casein, and phosphorylated JAK2 and STAT5 after Tyrphosting AG490 (30 μM) treatment for 48 h.
