# Supplementary material for: ELF5 modulates casein synthesis in goat mammary epithelial cells via JAK2/STAT5 signaling pathway
Source: Anim Biosci. 2025 Oct 22;39(2):250181. doi: 10.5713/ab.25.0181 (PMC12877387; doi:10.5713/ab.25.0181)
Supplement: Supplementary file 9 [file ab-25-0181-Supplementary-9.pdf]

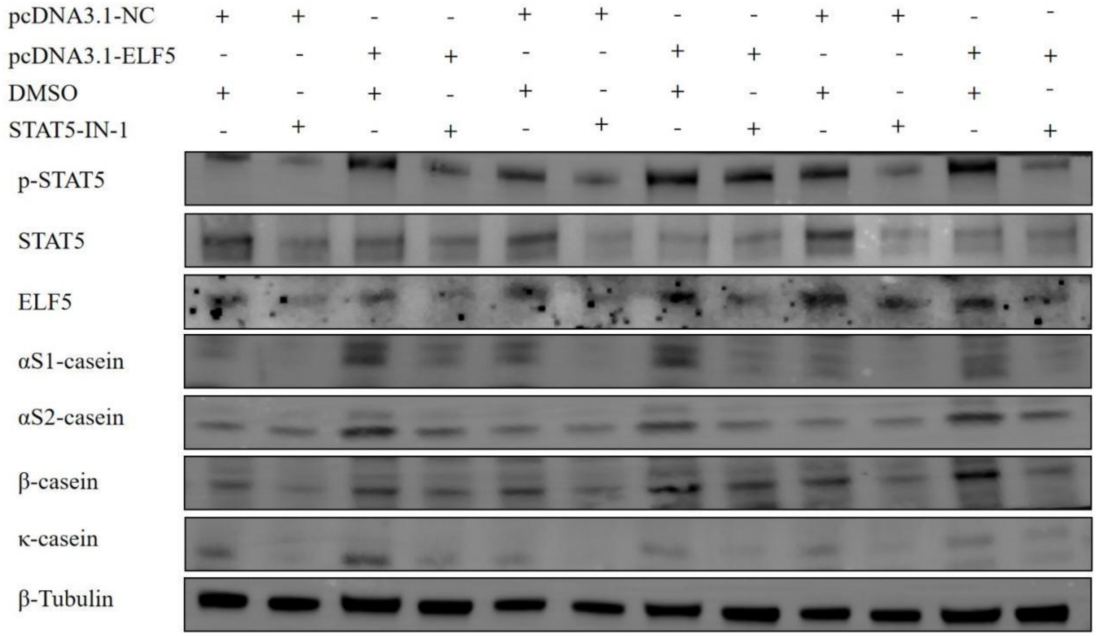


**Supplement 9.** The full Western blot image of Figure 7A. Cells were treated with STAT5-IN-1 (50 μM) or DMSO, followed by pcDNA3.1-ELF5 (or pcDNA3.1-NC) transfection for 48 h. The protein abundances of αS1-casein, αS2-casein, β-casein, κ-casein, and p-STAT5 were detected.
