# Supplementary material for: ELF5 modulates casein synthesis in goat mammary epithelial cells via JAK2/STAT5 signaling pathway
Source: Anim Biosci. 2025 Oct 22;39(2):250181. doi: 10.5713/ab.25.0181 (PMC12877387; doi:10.5713/ab.25.0181)
Supplement: Supplementary file 12 [file ab-25-0181-Supplementary-12.pdf]

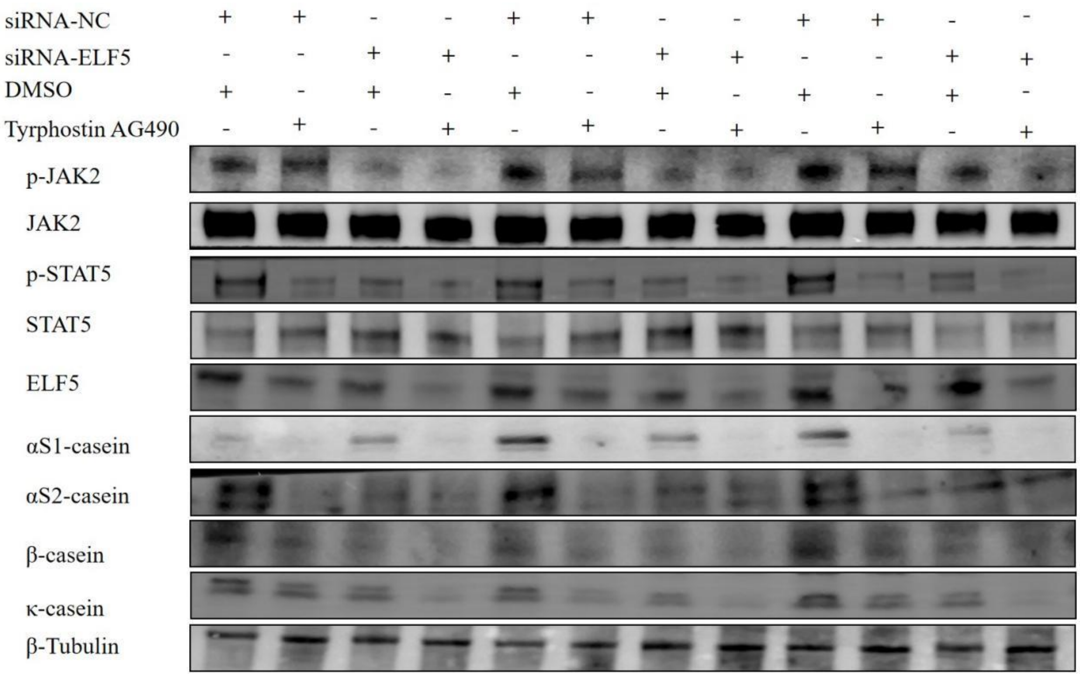

Supplement 12. The full Western blot image of Figure 8B. Cells were treated with Tyrphosting AG490 (30 μM) or DMSO, followed by siRNA-ELF5 (or siRNA-NC, 100 nM) transfection for 48 h. The protein abundances of αS1-casein, αS2-casein, β-casein, κ-casein, p-JAK2, and p-STAT5 were detected.
