# Supplementary material for: ELF5 modulates casein synthesis in goat mammary epithelial cells via JAK2/STAT5 signaling pathway
Source: Anim Biosci. 2025 Oct 22;39(2):250181. doi: 10.5713/ab.25.0181 (PMC12877387; doi:10.5713/ab.25.0181)
Supplement: Supplementary file 13 [file ab-25-0181-Supplementary-13.pdf]

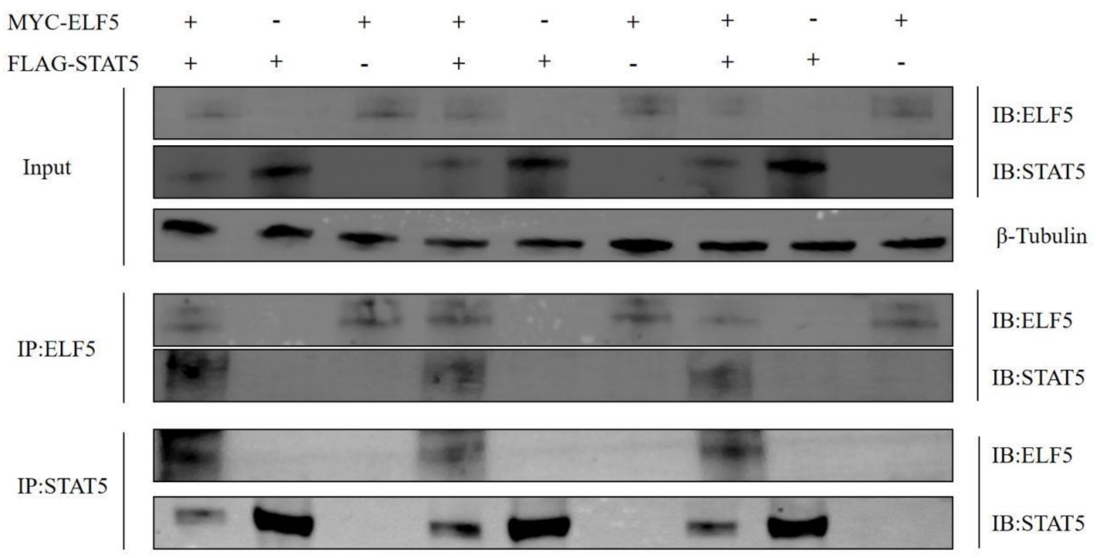

Supplement 13. The full Western blot image of Figure 9A. Cells were transfected with ELF5-MYC and STAT5-FLAG for 48 h. Then, total protein of cells was extracted and incubated with MYC (or FLAG) tag antibody and protein G magnetic beads at 4°C overnight. The protein abundance of ELF5 and STAT5 was detected.
